# Tolerising cellular therapies: what is their promise for autoimmune disease?

**DOI:** 10.1136/annrheumdis-2018-214024

**Published:** 2018-11-02

**Authors:** Chijioke H Mosanya, John D Isaacs

**Affiliations:** 1 Institute of Cellular Medicine, Faculty of Medical Sciences, Newcastle University, Newcastle upon Tyne, UK; 2 Musculoskeletal Unit, Newcastle upon Tyne Hospitals NHS Foundation Trust, Newcastle upon Tyne, UK

**Keywords:** Cellular therapies, tolerogenic dendritic cells, regulatory T-cells, mesenchymal stromal cells, TR1 cells, rheumatoid arthritis, type 1 diabetes, Crohn’s disease, multiple sclerosis, systemic lupus erythematosus, graft versus host disease, autoimmune thyroiditis, myasthenia gravis

## Abstract

The current management of autoimmunity involves the administration of immunosuppressive drugs coupled to symptomatic and functional interventions such as anti-inflammatory therapies and hormone replacement. Given the chronic nature of autoimmunity, however, the ideal therapeutic strategy would be to reinduce self-tolerance before significant tissue damage has accrued. Defects in, or defective regulation of, key immune cells such as regulatory T cells have been documented in several types of human autoimmunity. Consequently, it has been suggested that the administration of ex vivo generated, tolerogenic immune cell populations could provide a tractable therapeutic strategy. Several potentially tolerogenic cellular therapies have been developed in recent years; concurrent advances in cell manufacturing technologies promise scalable, affordable interventions if safety and efficacy can be demonstrated. These therapies include mesenchymal stromal cells, tolerogenic dendritic cells and regulatory T cells. Each has advantages and disadvantages, particularly in terms of the requirement for a bespoke versus an ‘off-the-shelf’ treatment but also their suitability in particular clinical scenarios. In this review, we examine the current evidence for these three types of cellular therapy, in the context of a broader discussion around potential development pathway(s) and their likely future role. A brief overview of preclinical data is followed by a comprehensive discussion of human data.

## Introduction

The complexity of immune tolerance mechanisms presents abundant opportunities for its breakdown, leading to the development of autoimmunity. In most cases, the precise pathogenesis of autoimmunity remains unknown but the genetic polymorphisms that underpin, for example, rheumatoid arthritis (RA), indicate that antigen presentation, cytokine dysregulation and the regulation of lymphocyte activation all play key roles. Furthermore, the clustering of different autoimmune diseases within families attests to common genetic predisposition and pathogenic mechanisms. However, for most autoimmune diseases, the provoking autoantigen(s) have not been defined and, critically, the predilection for the joint in RA versus the brain in multiple sclerosis (MS) versus the pancreas in diabetes mellitus remains enigmatic. Ultimately, the immune system can be viewed as a delicate balance of activation vs tolerance, with multiple mechanisms acting to maintain homeostasis.

Historically, management of autoimmune disorders involved managing end-organ manifestations such as insulin replacement in diabetes and control of pain and inflammation in conditions such as RA (table 1). During the second half of the 20th century the discovery of glucocorticoids and, subsequently, immunosuppressant medications enabled modification of the autoreactive process with reduced tissue damage and even improved life expectancy in diseases such as systemic lupus erythematosus (SLE). The 21st century has seen the biologics revolution with potent, targeted therapies that neutralise key proinflammatory cytokines or interfere with lymphocytes themselves. And, most recently, potent synthetic signalling pathway inhibitors are providing a further means to modulate immune reactivity.^1^ Nonetheless, current management options rarely lead to cure, or drug-free remission, and most patients require long-term maintenance therapy to control disease manifestations. For example, in RA, approximately 30% of patients achieve sustained remission, but 50% of these will flare if treatment is discontinued. The proportion that flare is usually higher once patients have moved on to more potent biological therapies.^2^ Because immunosuppressants downregulate the normal adaptive immune system, it is not surprising that several of the therapies in table 1 are associated with an enhanced infection risk, including opportunistic infections, and the development of malignancy. This is in addition to disease comorbidities and drug-specific side-effects, for example, with chronic glucocorticoids. In extreme cases, haematopoietic stem cell transplantation has been used to treat autoimmunity but, with rare exceptions, this intervention has not proved curative.^3 4^

**Table 1:** Current therapeutic options for management of autoimmunity

Therapy	Mode of action
Insulin, thyroxine, etc.	Replacement therapy
Paracetamol, opiates	Analgesia
Non-steroidal anti-inflammatory drugs: aspirin, ibuprofen, diclofenac, naproxen, etc.	Anti-inflammatory
COX-2 inhibitors: celecoxib, etc.	Anti-inflammatory
Glucocorticoids: prednisolone, prednisone, dexamethasone, etc.	Anti-inflammatory, immunosuppressive
DMARDS: MTX, sulphasalazine, leflunomide, hydroxychloroquine, azathioprine, mycophenolate mofetil, ciclosporin, etc.	Various, generally not well defined. Anti-inflammatory, immunosuppressive, possibly immunomodulatory. Some, such as MTX, may have more than one mode of action.
Cytokine blockade (anti-TNF, anti-IL6 receptor)	Anti-inflammatory and immunosuppressive, immunomodulatory
B-cell depletion/modulation (anti-CD20, anti-BLyS)	Immunosuppressive, immunomodulatory
Costimulation blockade (abatacept)	Immunosuppressive, immunomodulatory
Janus kinase inhibitors (tofacitinib, baricitinib, others in development)	Anti-inflammatory, immunosuppressive, immunomodulatory
Intravenous immunoglobulins	Immunomodulatory (via Fc receptor interactions)
Plasmapheresis	Immunosuppressive, immunomodulatory (by removing (auto)antibodies and other soluble mediators)

For several therapies, particularly DMARDs, the precise mode of action is not known. Immunomodulation denotes that the treatment has a specific and defined effect on the immune system.

DMARDs, disease-modifying anti-rheumatic drugs; MTX, methotrexate.

The holy grail of treatment for autoimmunity would be the reinstatement of immune tolerance. So-called therapeutic tolerance induction offers the opportunity to ‘reset’ the diseased immune system to a state of immune tolerance, theoretically providing for long-term, drug-free remission.^5^ While multiple strategies have proven effective in animal models of autoimmunity and transplantation, translation to the clinic has been slow. Multiple explanations have been offered, relating to disease stage, therapeutics employed, and the need for better biomarkers of tolerance, among others. Nonetheless, because of the slow progress with therapeutics that target the immune system, such as biologic drugs and peptides, recent strategies have focused on the use of tolerogenic cells themselves.

## Tolerogenic cell types

In recent years, investigators have turned their attention to the ex-vivo expansion or differentiation of ‘tolerogenic’ immune cells, followed by their adoptive transfer, as a potential route to therapeutic tolerance induction. To a large degree, these strategies have been catalysed by advances in bio-manufacturing in general, with robust and scalable processes leading to the efficient manufacture of advanced cellular therapies.^6^ To date, three main types of tolerogenic cell have been the focus of therapeutic strategies in humans.

### Mesenchymal stromal cells

**Figure 1:**
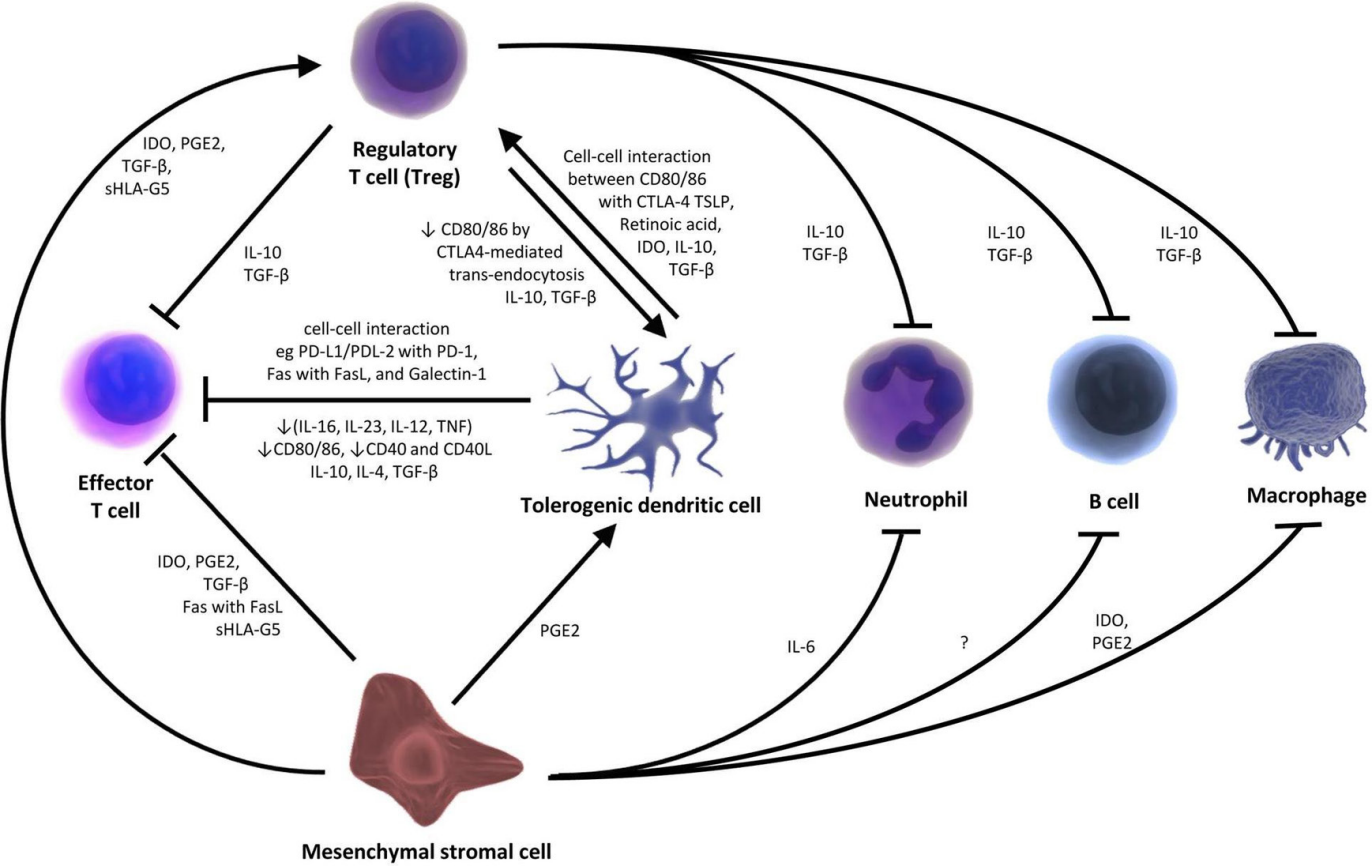
A schematic representation of the mechanisms of action of tolerogenic cells. MSCs promote the differentiation and survival of Tregs and tolDC. Tregs and tolDC, on the other hand, enjoy a mutual bidirectional positive interaction with each other. Tregs and MSCs inhibit the actions of B cells, effector T cells, macrophages and neutrophils through cell-cell contact (eg, Fas:Fas Ligand (FasL) mediated deletion), and various soluble factors such as TGF-β, IDO, PGE2, IL-10, IL-6, and sHLA-G5. MSCs also act through extracellular vesicles.^8–10 18^ TolDC directly inhibit effector T cells through various mechanisms. These include: cell-cell ligand-receptor mediated deletion, for example, Fas: FASL, PD-L1 and PD-L2 on tolDC and PD-1 receptors on effector T cells; effector T cell anergy secondary to low expression of co-stimulatory molecules CD80/CD86, CD40 and pro-inflammatory cytokines (TNF, IL-12, IL-21 and IL-16) by tolDC. Other mechanisms include soluble anti-inflammatory cytokines such as IL-10, IL-4 and TGF-β.^26 27^TolDC directly promote Tregs and so indirectly inhibit other immunogenic cells through Tregs. Mechanisms include soluble factors such as IL-10, IDO, TGF-β and TSLP and cell-cell interaction between CTLA-4 and CD80/86. This interaction, in turn, leads to transendocytosis of CD80/86 and further tolerogenic phenotypic ‘reinforcement’ of tolDC. Tregs also promote tolDC via IL-10 and TGF-β.^26 27^ CTLA-4, cytotoxic T-lymphocyte associated protein 4; IDO, indoleamine-2,3-dioxygenase; IL, interleukin; MSCs, mesenchymal stromal cells; PDL, programmed death ligand; PGE2, prostaglandin E2; sHLA, soluble human leucocyte antigen; TGF-β, transforming growth factor beta; tolDC, tolerogenic dendritic cells; TSLP, thymic stromal lymphopoietin.

Mesenchymal stromal cells (MSCs) are spindle-shaped, plastic-adherent, progenitor cells of mesenchymal tissues with multipotent differentiation capacity.^7^ MSCs can modulate innate and adaptive immune cells including dendritic cells (DC), natural killer cells (NK) cells, macrophages, B-lymphocytes and T-lymphocytes. This occurs via both cell-cell contact and paracrine interactions through several soluble mediators including indoleamine-2,3-dioxygenase (IDO), prostaglandin E2 and transforming growth factor β.^8–10^ These and other mechanisms have been summarised in figure 1. By definition, MSCs can differentiate into bone, chondrocytes and adipose tissue in vitro; they are phenotypically positive for CD105, CD73 and CD90 and negative for haematopoietic markers CD45, CD34, CD14, CD11b, CD3 and CD19.^7 11^ They do not express Class II MHC molecules unless stimulated by interferons^7^ and lack costimulatory molecules such as CD40, CD80 and CD86.

**Figure 2:**
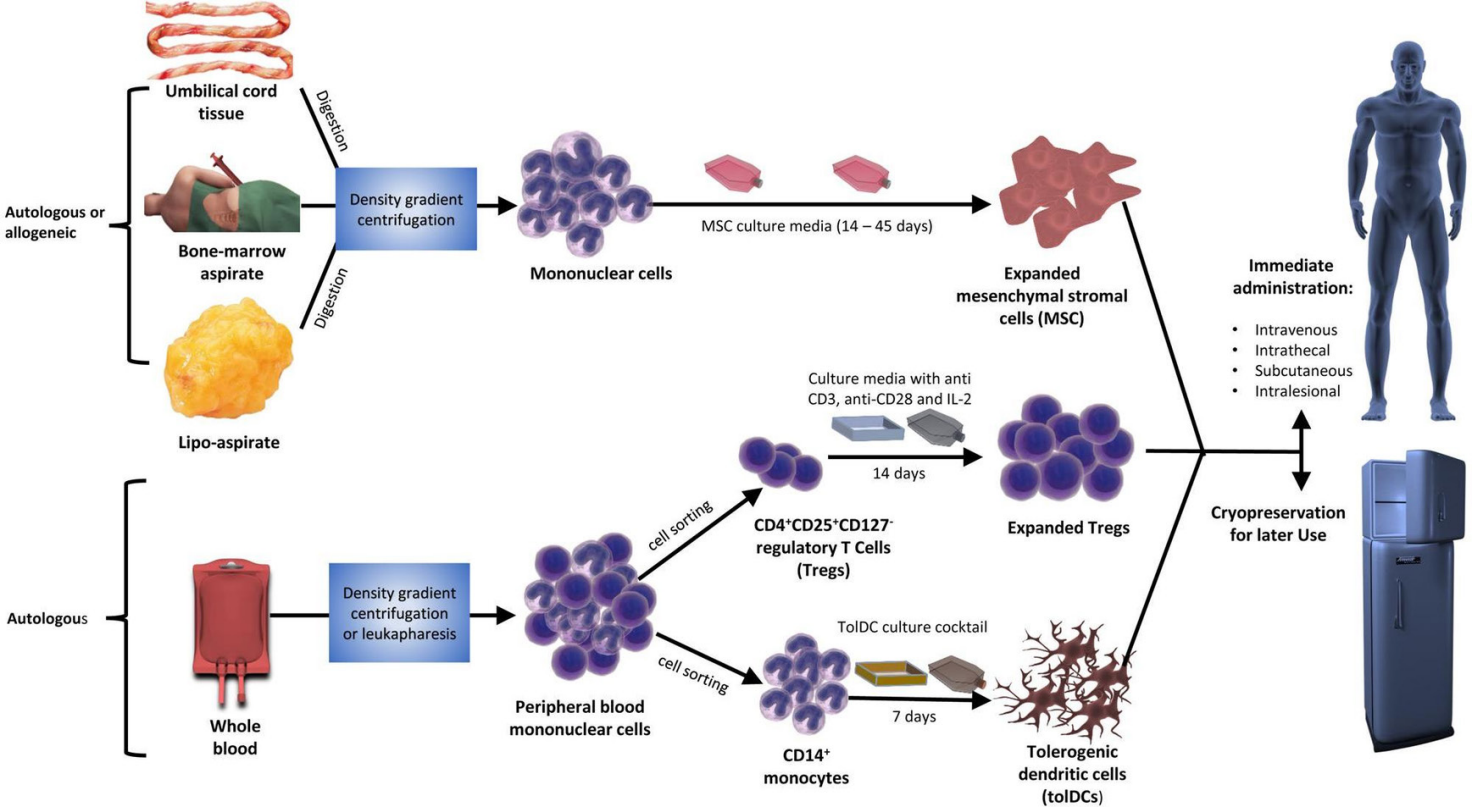
Preparation and administration of tolerogenic cellular therapies. This figure describes the process of cellular therapy manufacture and administration. Sources of substrate cells include autologous or allogeneic umbilical cord tissue, bone marrow aspirate and lipo-aspirate for mesenchymal stromal cells and autologous whole blood for expanded regulatory T cells and tolerogenic dendritic cells. Mononuclear cells are usually extracted by density gradient centrifugation of whole blood, bone marrow aspirate and digested tissue (lipo-aspirate and umbilical cord tissue) or by leukapheresis (whole blood). Mononuclear cells are then cultured in the appropriate media and culture conditions for the requisite duration or number of passages. Harvested cells can be administered immediately through various routes (subcutaneous, intravenous, intralesional and intrathecal) or cryopreserved for future use.

Exposure to proinflammatory cytokines IFN-γ, TNF and IL-1β^10^ and activation by exogenous/endogenous danger signals such as bacterial products and heat shock proteins through Toll-like receptor 3 (TLR3) ‘licenses’ MSCs to become immunosuppressive^12^; in contrast, activation through TLR4 confers a proinflammatory signature and, under some conditions, TLR3 signals may do the same.^12 13^ The immunomodulatory functions of MSC include their ability to: inhibit T cell proliferation and promote their differentiation into regulatory T cells (Tregs);^14^ inhibit the CD4^+^ T cell induced differentiation of B-cells into plasma cells and directly inhibit B-cell proliferation, differentiation and chemotaxis.^15^ Although MSCs reside in most postnatal organs and tissues,^16^ they are readily harvested from bone marrow, adipose tissues, umbilical cord blood and Wharton’s jelly (figure 2).

### Tolerogenic dendritic cells (tolDC)

DCs are best recognised for their antigen presenting functions in driving immune responses against pathogens and tumour cells. However, DC also play crucial roles in co-ordinating central and peripheral tolerance processes, such that absent or deficient DC associate with an increased tendency to develop autoimmunity.^17 18^ Furthermore, in autoimmunity, DC are skewed to a proinflammatory state, producing more proinflammatory cytokines and leading to activation and differentiation of autoreactive T cells.^19^

Immature DC are usually regarded as tolerogenic, whereas mature DC can exert either tolerogenic or immunogenic functions depending on signals received during maturation from the microenvironment and invading pathogens. For instance, bacterial lipopolysaccharides induce immunogenic maturation of DC by upregulating surface MHC complexes and T cell costimulatory molecules (CD80, CD86),^20 21^ while schistosomal lysophosphatidylserine, anti-inflammatory cytokines (eg, IL-10) and glucocorticoids induce a tolerogenic phenotype.^18^ Tolerogenic dendritic cells (tolDC) induce peripheral tolerance by induction of anergy and deletion of T cells,^22^ blockade of T cell expansion^23^ and induction of regulatory T cells (Tregs).^24 25^ Tregs in turn induce the regulatory properties of DC (figure 1). These mechanisms have already been reviewed.^26 27^

Several methods can be used to produce stable tolDC ex vivo, with limited or no capacity to transdifferentiate into immunogenic DC. Common methods include inhibiting the expression of immune-stimulatory molecules (CD80/CD86 and IL-2)^28–30^ or stimulating constitutive expression of immunosuppressive molecules such as IL-4, IL-10 and CTLA-4,^31–35^ through genetic engineering. Also, exposing differentiating DC ex-vivo to drugs such as dexamethasone and vitamin D3^36 37^ or immunosuppressive cytokines such as IL-10 and TGF-β^38–40^ and lipopolysaccharides^41^ can be used to produce tolDC. These and other methods have been extensively reviewed elsewhere.^42^

### Regulatory T cells (Tregs)

Tregs are a subset of T cells expressing CD4, CD25 and intracellular Forkhead Box P3 (FoxP3) protein that inhibit the functions of effector T cells as well as other immune effector cells and so are essential for immune tolerance.^43 44^ They mediate their effects by producing immunosuppressive cytokines and by cell-to-cell contact, following stimulation via their antigen-specific T cell receptors (TCR). These mechanisms also modulate other immune responses in an antigen-non-specific manner through ‘bystander suppression’ and ‘infectious tolerance’.^45 46^ Treg depletion and dysfunction have been implicated in a variety of autoimmune disorders including type 1 diabetes, RA, SLE and, classically, with an inherited deficiency of FoxP3, immune dysregulation polyendocrinopathy enteropathy X linked syndrome.^47 48^ These findings support the possibility that ex-vivo expansion and transfusion of autologous or allogeneic Tregs could provide an effective therapeutic strategy for unwanted immunopathology such as autoimmunity.

In the past, the lack of reliable Treg surface markers and the resultant possibility of simultaneously isolating and transfusing proinflammatory T cells slowed the development of protocols for Treg isolation and expansion.^5^ More recent studies have used CD4, CD25 and CD127 cell surface markers to isolate CD4^+^CD127^lo/-^CD25^+^ Tregs from blood.^49 50^ Other types of regulatory T cells exist, such as T-regulatory type 1 (Tr1) cells, which secrete IL-10.^51^ These are a distinct population of regulatory T cells that only transiently express FoxP3, on activation.^52^ They coexpress CD49b and LAG-3, and secrete high levels of IL-10 but low amounts of IL-4 and IL-17. Suppression is dependent on IL-10 and TGF-β and they kill myeloid antigen-presenting cells via granzyme B release.

### Migration of tolerogenic cells

MSCs, Tregs and tolDC express a host of homing receptors that are important for their transmigration from the tissue of administration (eg, skin or vascular system) to activation sites (eg, regional lymph nodes) and, ultimately, to the target organs. For instance, FoxP3+ Tregs express CC receptor 7 (CCR7), CCR4, CCR6, CXC receptor 4 (CXCR4) and CXCR5. They also express CD103 (integrin α_E_β_7_) (whose ligand is E-cadherin expressed by epithelial cells) and CD62L (L-selectin) (whose ligands are the lymph node and mucosal lymphoid tissue endothelial cell addressins CD34, GlyCAM-1 and MAdCAM-1).^53^ Activated tolDC express CCR7 and migrate to CC chemokine ligand 19 (CCL19),^54^ underpinning migration to regional lymph nodes. MSCs, on the other hand, express a restricted set of chemokine receptors (CXCR4, CX3CR1, CXCR6, CCR1, CCR7) and have shown appreciable chemotactic migration in response to the chemokines CXC ligand 12 (CXCL12), CX3CL1, CXCL16, CCL3 and CCL19.^55^ MSCs may also exert tolerogenic effects in distant tissues via extracellular vesicles.^10^ It is clearly important that migration potential is considered during the generation of cellular therapies.

## Cellular therapies for therapeutic tolerance

### What could cellular therapies achieve?

Numerous preclinical studies using animal models of autoimmune disorders have shown potent tolerogenic effects of these various immune modulatory cells, although some mechanisms of action remain unclear. Animal models do not faithfully replicate all mechanisms of human autoimmunity but positive results have provided the scientific basis to catalyse clinical trials.

### Mesenchymal stromal cells (MSCs)

The first ever preclinical study of MSCs in an autoimmune setting was in experimental auto-immune encephalomyelitis (a model for MS).^56^ MSCs were effective in treating the disease and were shown to be strikingly effective if injected before or at the onset of disease. Further studies in experimental MS buttressed this finding^57–60^ and showed that MSCs control disease through inhibition of CD4^+^ Th17 T cells,^58^ generation of CD4^+^CD25^+^FoxP3^+^ Tregs^60^ and through hepatocyte growth factor production.^59^ Therapeutic efficacy was also observed in the MRL/Lpr^61^ and NZB/W F1^62 63^ mouse models of SLE. MSCs were effective in collagen-induced arthritis,^64 65^ Freund’s adjuvant-induced arthritis and K/BxN mice with spontaneous erosive arthritis.^66^ These studies have been reviewed elsewhere.^10^

Results from early clinical trials in MS showed good tolerability and some potential efficacy^67–70^ (table 2A) associated with increased number of Tregs in the peripheral blood of patients.^67^ In the most recent controlled study,^70^ 13 patients received MSCs while 10 patients received conventional MS treatment. The active treatment group showed a more stable disease course and a transient increase in immunomodulatory cytokines. A placebo-controlled dose-ranging study of mesenchymal-like cells derived from placenta in patients with MS^71^ used a distinct type of cell with immunomodulatory and regenerative properties, which do not fully meet ISCT criteria for MSCs (and therefore not included in table 2A). Their phenotype includes CD10+, CD105+ and CD200+; they are CD34- and, like MSCs, do not express class II HLA or costimulatory molecules CD80, CD86. The cells appeared safe and well tolerated in patients with relapsing remitting MS and secondary progressive MS.

**Table 2A:** Clinical trials of mesenchymal stromal cells in MS, RA and SLE

Diseases and clinical trials	Number of patients, source of cells, dose and route of administration	Outcomes	Comments
Multiple sclerosis, MS			
1. Karussis *et al* (2010)^67^ Phase I/II uncontrolled feasibility study of patients with MS and ALS	34 patients (15 with MS, 19 with ALS) received autologous BM-derived MSCs intrathecally (n=34) at a mean dose of 63.2×10^6^ in 2mls of saline and intravenously (n=14) at a mean dose of 23.4×10^6^ cells in 2mls of saline.	No major AEs. EDSS score improved over 6 months. Proportion of CD4^+^CD25^+^ Tregs increased, and expression of CD40, CD83, CD86 and HLA-DR on myeloid dendritic cells decreased 24 hours post-administration. MRI of MSC labelled with superparamagnetic particles showed MSCs in meninges, subarachnoid space, and spinal cord.	No comparison between intravenous and Intrathecal routes as regards homing of MSCs to the CNS. Cryopreserved cells were used.
2. Bonab *et al* (2012)^68^ Phase II uncontrolled study of patients with SPMS	22 patients received Intrathecal, autologous BM-derived MSCs at a mean dose of 29.5×10^6^ cells in 10mls of normal saline.	AEs were low-grade: transient fever, headache, nausea/vomiting (related to lumbar puncture). Disease progression stabilised in the short-term evidenced by MRI and EDSS score.	After initial improvement some patients reported worsening EDSS, and about 25% showed worsening lesions on MRI, after 12 months. Cryopreservation was not discussed.
3. Connick *et al* (2012)^69^ Phase IIa feasibility/ proof-of-concept study in patients with SPMS	10 patients received autologous bone marrow (BM)-derived MSCs intravenously at a mean dose of 1.6×10^6^ cells/kg.	Mild AEs such as transient post-transfusion rash and self-limiting bacterial infections. Improvement in visual acuity, visual evoked potentials, optic nerve area and EDSS. No change in post-treatment T cell subset counts.	Cryopreserved cells were used.
4. Li *et al* (2014)^70^ Randomised Controlled Phase II study in patients with RRMS and SPMS	13 patients received 3 cycles of intravenous, allogeneic umbilical cord (UC)-derived MSCs, 2 weeks apart, at a dose of 4×10^6^ cells/kg body weight in 100mls of saline. Conventional treatment (anti-inflammatory and immunosuppressants) was continued; 10 patients received only conventional treatment.	Reduced frequency of recurrence in the treatment group, who also had a more steady disease course. No significant adverse event. Transient improvement in immunomodulatory cytokines was recorded	Randomised controlled study but not blinded. Cryopreservation was not discussed
Rheumatoid arthritis			
5. Wang *et al* (2013)^72^ Phase II non-randomised, controlled study	172 patients with active RA. 136 received 4×10^7^ allogeneic UC-derived MSCs in 40mls of intravenous saline while 36 received only saline. All patients continued their DMARDS.	No serious adverse events. TNF-alpha and IL-6 decreased while FoxP3^+^ Tregs increased in the treatment group after infusion. Better clinical outcomes (ACR responses, HAQ and DAS28) after 3 months in the treatment group	Non-randomised study. Treatment group and control group were recruited in different time frames. Cryopreserved cells were used
6. Alvaro-Gracia *et al* (2017)^73^ Dose-escalation, randomised, single-blind (double-blind for efficacy), phase Ib/IIa study	53 patients with refractory RA (failed two biologics) received three intravenous infusions at different doses (1×10^6^, 2×10^6^ and 4×10^6^ cells/kg) of allogeneic, adipose-derived MSCs or placebo	Generally well-tolerated. Mild adverse events. Dose-dependent response especially DAS28-ESR at 1 month and 3 months post-infusion. Distribution of T cell populations was not significantly modified.	First placebo-controlled study of MSCs in RA. 19% of patients generated mesenchymal stromal cell-specific anti-HLA1 antibodies without apparent clinical consequences. Cryopreserved cells were used
SLE			
7. Sun *et al* (2009)^74^ Safety of MSC in Patients with refractory SLE	Four patients with refractory SLE received intravenous, allogeneic BM-derived MSCs at a dose of 1×10^6^ cells/kg.	Safe and well-tolerated. Stable course of SLE disease activity by 12–18 months post-treatment, with improvement in SLEDAI and serological markers.	First study in SLE. Provided evidence for further studies in SLE. Cryopreservation was not discussed.
8. Liang *et al* (2010)^75^ Early phase safety/efficacy study in refractory SLE	15 patients with refractory SLE were treated with one intravenous infusion of 1×10^6^ cells/kg allogeneic BM-MSC. Mean follow-up period of 17.2 months	No serious adverse events. All patients clinically improved with decrease in SLEDAI, proteinuria, and anti-dsDNA.	Improvement in some patients allowed reduction in doses of steroids and immunosuppressants. Cryopreservation was not discussed.
9. Sun *et al* (2010)^76^ Early phase I/II study	16 patients with active and refractory SLE on different treatment regimens received 1×10^6^ cells/kg intravenous of UC-derived MSC.	Significant improvement in SLEDAI score, autoantibodies, complement C3 and renal function accompanied by increased Tregs.	Patients clinically improved despite reducing doses of maintenance steroids and immunosuppressants. Cryopreservation was not discussed.
10. Wang *et al* (2012)^77^ Early phase I/II study. Compared the efficacy of single and double infusions	58 patients with refractory and active SLE. 30 received one intravenous dose of 1×10^6^ cells/kg allogeneic BM-MSCs or UC-MSCs, while 28 received two infusions of 1×10^6^ cells/kg 1 week apart.	No remarkable difference in SLEDAI and serological marker changes between the two groups.	Non-significance of difference in clinical improvement between single and double dose cohorts may be related to sample size. Cryopreservation was not discussed.
11. Li *et al* (2013)^78^ Early phase I/II study in patients with SLE with refractory cytopaenia	35 patients with SLE with refractory cytopaenia received 1×10^6^ cells/kg of either allogeneic BM-derived or allogeneic UC-derived MSCs and followed up for an average of 21 months.	Well-tolerated. Significant improvement in blood cell counts after MSC treatment. Clinical improvement was also associated with increased Tregs and decreased Th17.	Focused on haematological parameters in SLE. Cryopreservation was not discussed.
12. Wang *et al* (2013)^79^ Early phase I/II 4 year single-centre study	87 patients with SLE. Allogeneic BM-MSC or UC-MSC infused intravenously at 1×10^6^ cells/kg. Some patients were treated with cyclophosphamide to inhibit active lymphocyte response. 18 patients received repeat doses of MSC for relapses	Generally safe and well-tolerated. SLEDAI score, renal function and blood counts significantly improved for up to 4 years. All patients underwent tapering of steroids and immunosuppressants according to clinical status.	No differences in outcomes between those pretreated with cyclophosphamide and those that were not. No differences with regard to source of cells (UC and BM). Cryopreservation was not discussed.
13. Wang *et al* (2014)^80^ Multicentre phase I/II study	40 patients with active and refractory SLE received two intravenous doses of 1×10^6^ cells/kg allogeneic UC-derived MSCs while still maintaining baseline immunosuppressants+/-steroids.	Well-tolerated. 60% achieved major clinical response or partial clinical response as determined by BILAG scores. SLEDAI, renal function and serological indices also improved allowing tapering of steroid and immunosuppressant doses.	12.5% and 16.7% relapse rate at 9 and 12 months, respectively. Cryopreservation was not discussed.

ACR, American College of Rheumatology; AE: adverse events; ALS, amyotrophic lateral sclerosis; BM, bone marrow; BILAG, British Isles Lupus Activity Group; DAS28, Disease Activity Score-28 joint count; EDSS, Expanded Disability Status Score; HAQ, Health Assessment Questionnaires; RA, rheumatoid arthritis; RRMS, relapsing remitting multiple sclerosis; SLEDAI, Systemic Lupus Erythematosus Disease Activity Index; SPMS, secondarily progressive multiple sclerosis; UC, umbilical cord.

In RA, MSCs were well-tolerated and showed preliminary efficacy with improvements in clinical outcomes when combined with disease-modifying anti-rheumatic drugs (DMARDS).^72 73^ In the first placebo-controlled randomised trial of MSCs in RA,^73^ 40 patients who had failed at least two biological DMARDS received intravenous infusions of adipose-derived MSCs at varying dose, while 7 patients received placebo. Adverse events were few and included fever and respiratory tract infections; however, serious adverse events included a lacunar infarction. Clinical outcomes, especially DAS28-ESR, showed a dose-dependent improvement.

The first case series of MSC in patients with SLE was published in 2009.^74^ Four patients with cyclophosphamide/glucocorticoid-refractory SLE were treated with bone marrow-derived MSCs. After 12–18 months of follow-up, all showed improvement in disease activity, renal function and serological markers. Subsequent studies, mainly by the same group, have confirmed that MSCs are safe in SLE and reported promising results such as improvement in renal function, proteinuria, SLE disease activity indices, anti-dsDNA titre and circulating Tregs.^75–80^ In the most recent multicentre study, up to 60% of treated patients achieved either major or partial clinical response as determined by British Isles Lupus Activity Group scores.^80^ However, a relapse rate of 12.5% at 9 months may warrant repeated infusions of MSCs. An analysis, by the same group, of four patients with diffuse alveolar haemorrhage in SLE using high resolution CT scan showed resolution of lung pathology after treatment with MSCs.^81^

A serious complication of Crohn’s disease is perianal fistulae. MSCs have been extensively studied in Crohn’s disease for their immunomodulatory properties and for their ability to differentiate into mesodermal tissues with tissue repair capabilities (table 2B). Results in Crohn’s disease are encouraging with patients who received MSCs experiencing significant improvement in fistulae while reporting just minor side effects.^82–90^ The unprecedented success of MSCs in a recently concluded phase III multicentre clinical study in Crohn’s disease across seven European countries and Israel implies that MSCs could become a treatment of choice for Crohn’s fistulae refractory to conventional treatment. In this study,^90^ 212 patients with Crohn’s disease-associated fistulae received intralesional injections of either MSCs or placebo. Fifty per cent of the treatment group achieved combined clinical and radiological remission at 24 weeks compared with 34% of the placebo group, with only minor adverse effects reported. MSC have also been successfully embedded in an absorbable biomaterial and surgically delivered for the treatment of fistulae associated with Crohn’s disease.^91^ In this study, 12 patients safely received MSC embedded in a Gore fistula plug with fistula healing rate of 88.3% at 6 months.

**Table 2B:** Clinical trials of mesenchymal stromal cells in Crohn’s disease

Diseases and clinical trials	Number of patients, source of cells, dose and route of administration	Outcomes	Comments
Crohn’s disease			
1. Garcia-Olmo *et al* (2005)^82^ Phase I study	5 patients with fistulating Crohn’s disease received intralesional injections of autologous adipose derived at a dose of between 3 to 30×10^6^ cells depending on yield.	Six out of eight fistulae healed completely after 8 weeks. No adverse effects	First clinical trial of mesenchymal stem cells to treat Crohn’s disease. Cells were not cryopreserved. Study published before the ISCT criteria for MSC was set so cells were not assessed against the ISCT criteria.
2. Garcia-Olmo *et al* (2009) 83 Phase II multicentre randomised controlled trial	49 patients with complex fistulae. 24 received intralesional injection of 20×10^6^ cells/kg allogeneic adipose derived stem cells; 25 received fibrin glue.	Significantly better fistula healing in the treatment group (relative risk 4.43). Quality of life scores were also higher in the treatment group	
3. Duijvestein *et al* (2010)^84^ Phase I study	9 patients with refractory Crohn’s disease received two IV infusions of 1–2×10^6^ cells/kg autologous BM-derived MSCs 7 days apart.	Well tolerated with few mild adverse events such as allergic reaction in a patient. Three patients showed improvement in Crohn’s disease activity indices 6 weeks post-treatment	Three patients required surgery due to worsening disease.
4. Ciccocioppo *et al* (2011)^85^ Phase I/II study in patients with fistulating Crohn’s disease	10 patients with refractory Crohn’s disease received intralesional injection of autologous BM-derived MSCs at a median dose of 20×10^6^ cells every 4 weeks for a median four cycles (injections were stopped when patients achieved remission or exhausted supplies of autologous MSCs).	Clinical improvement in all patients with seven achieving complete fistula closure and three achieving partial closure. Few adverse events were documented. Tregs also increased post-treatment and remained stable post follow-up.	Cryopreserved cells were used
5. Liang *et al* (2012)^86^ Use of MSCs in inflammatory bowel diseases	7 patients with inflammatory bowel disease (4 Crohn’s, three ulcerative colitis) received IV infusion of allogeneic BM-derived or UC-derived MSCs at a dose of 1×10^6^ cells/kg.	Five patients achieved clinical remission at 3 months. Endoscopic improvement (assessed by endoscopic index of severity score) was also observed in three patients.	Cryopreservation was not discussed
6. de la Portilla *et al* (2013)^87^ Phase I/IIa multicentre study	24 patients received intralesional injections of allogeneic adipose derived stem cells at a dose of 20×10^6^ cells.	More than half of patients showed healing of fistulae at 6 months. Up to 30% had complete fistula closure	Cryopreserved cells were used
7. Forbes *et al* (2014)^88^ Phase II open-label multicentre study	16 patients with refractory Crohn's disease received IV infusion of allogeneic MSCs at a dose of 2×10^6^ cells/kg weekly for 4 weeks.	Safe and well-tolerated. Clinical improvement observed in at least 12 patients, 8 of whom achieved clinical remission 42 days post-infusion.	Cryopreserved cells were used
8. Molendijk *et al* (2015)^89^ Phase I/II double-blind, placebo-controlled, dose-escalating study	21 patients with refractory fistulating Crohn’s disease received intralesional injection of 1×10^7^ or 3×10^7^ or 9×10^7^ allogeneic BM-derived MSC or placebo.	Well tolerated. More significant fistulae healing in all dosing groups when compared with placebo. Most observed with 3×10^7^ dose.	Expanded half-products were cryopreserved until needed. Two weeks before treatment, they were thawed and further expanded to yield sufficient numbers of cells.
9. Panés *et al* (2016)^90^ Phase III randomised, double-blinded controlled study	212 treatment- refractory Crohn’s disease patients with fistulae. 107 Patients received 120×10^6^ allogeneic adipose derived MSCs as a single intralesional dose, while 105 received placebo (saline).	Significantly greater remission rates in the treatment group compared with the placebo group. Few adverse events notably proctalgia and anal abscess.	First phase III study. Effective treatment option for Crohn’s disease patients that have failed conventional treatment options. Cryopreserved cells were used
10. Dietz *et al* (2017)^91^ Phase I trial of autologous stem cells applied in a bio-absorbable matrix	12 patients with fistula secondary to Crohn’s disease received autologous adipose-derived MSC embedded in a Gore Bio-A Fistula Plug through surgical insertion at a mean dose of 20×10^6^ per plug	Procedure was safely tolerated and few adverse events were reported. 75% of patients achieved complete healing at 3 months, while 83.3% achieved fistula closure at 6 months.	Cryopreserved cells were used. Thawed cells were reincubated with a fistula plug in a polypropylene coated bioreactor for 3–6 days prior to surgery. This is the first combination of mesenchymal stromal cells in a biomaterial for local application in Crohn’s disease.

BM, bone marrow; MSCs, mesenchymal stromal cells; UC, umbilical cord.

MSCs have also been used in several trials to prevent and treat graft versus host disease (GVHD). In a multicentre phase II study, 55 patients with steroid resistant severe acute GVHD received MSCs at a median dose of 1.4×10^6^ cells, obtained either from HLA-identical sibling donors, haploidentical donors or third-party HLA-mismatched donors. Up to 30 patients achieved complete clinical response independent of cell source.^92^ In a recent phase II study, prophylactic MSCs were successfully used to prevent GVHD following HLA-haploidentical stem cell transplantation.^93^

A potential advantage of MSC therapy over some other tolerogenic therapies is that their lack of MHC class expression means that they can be derived from either an autologous or allogeneic source with little or no risk of immune rejection.^10^ Thus, cryopreserved allogeneic MSC could become an ‘off-the-shelf’ therapy rather than a bespoke therapy requiring preparation at the point of delivery. In tables 2A and 2B, the source of MSC is indicated for each trial listed.

### Tolerogenic dendritic cells (tolDC)

In an early murine experiment, allogeneic DC transfer from diabetic non-obese diabetic (NOD) mice to prediabetic NOD mice prevented development of diabetes in the latter.^94^ The hypothesis was that the diabetic NOD mice DC contained pancreatic antigens that conferred immunoregulatory properties, possibly by targeting regulatory T cells specific to those antigens. Since then, many preclinical studies have demonstrated that ex vivo generated DC, with an anti-inflammatory or tolerogenic phenotype, can effectively suppress or ‘switch off’ auto-immune disorders such as diabetes,^95 96^ arthritis,^97^ MS,^98 99^ autoimmune thyroiditis^100^ and myasthenia gravis.^39^ In most studies, tolDC were pulsed with antigens to confer specificity: bovine serum albumin for bovine serum albumin-induced arthritis,^97^ pancreatic islet lysate for diabetes,^95^ encephalitogenic myelin basic protein peptide 68–86 (MBP 68–86) for MS^99^ and thyroglobulin for autoimmune thyroiditis.^100^ Interaction of autoreactive T cells with such partially mature or ‘deviated’ DC results in their loss of functionality (anergy), apoptosis or acquisition of regulatory function. The majority of the studies aimed at prevention of autoimmunity by administering tolDC in the predisease state (either prophylactically or immediately post-immunisation).^39 95 96 100^ However, tolDC also arrested established disease,^39 41 97^ with similar outcomes to prophylactic models.^98^ These studies have been summarised elsewhere.^42^

The first clinical trial of tolDC in a human autoimmune disorder was in type 1 diabetes^101^ (table 3). In this study, 10 million autologous DC were safely administered intradermally into patients two times a week for a total of 4 doses, without serious adverse effects. Two forms of DCs were used: immature ‘control DC’ cultured from monocyte precursors using IL-4 and GM-CSF and immunosuppressive DC (iDC) genetically manipulated ex-vivo to block the expression of costimulatory molecules CD80/CD86.^101^ TolDC were not loaded with autoantigens in this trial. Some therapeutic efficacy was suggested as some patients showed elevated c-peptide levels post-treatment, indicative of increased endogenous insulin production. In a phase I single centre study, tolDC were also safely infused intraperitoneally in patients with refractory Crohn’s disease and showed some potential efficacy.^102^ Other studies of TolDC in autoimmunity are in inflammatory arthritis: the AuToDeCRA study where autologous tolDC were loaded with autologous synovial fluid as a source of autoantigen^103^ and the Rheumavax study where autologous tolDC were exposed to citrullinated peptides to confer antigen specificity and administered intradermally to patients with RA.^104^ In the phase I AuToDeCRA study, DC were injected arthroscopically into an inflamed knee joint, as a robust test of their stability and safety in an inflamed environment. There was no evidence that the procedure provoked a flare of symptoms. In a study published only as an abstract, recombinant autoantigen-loaded tolDC were administered subcutaneously to patients with RA at doses of 0.5×10^7^ and 1.5×10^7^ cells. Dose-dependent efficacy was reported, especially in autoantigen positive patients and autoantibody titres also decreased.^105^ Other trials in Crohn’s disease, RA and MS are ongoing and results are yet to be published.^27^

**Table 3:** Clinical trials of TolDC in autoimmune disorders

Diseases and clinical trials	Number of patients, source of cells, dose and route of administration	Outcomes	Comments
Diabetes mellitus			
1. Giannoukakis *et al* (2011)^101^ A randomised double-blind phase I study	10 patients with type 1 diabetes received 10×10^6^ autologous peripheral blood-derived dendritic cells intradermally every 2 weeks for 4 administrations (7 received ex vivo manipulated DC lacking CD80/CD86 while 3 controls received non-manipulated immature DCs).	Safely tolerated. Significant increase in the proportion of B220^+^ CD11c^-^ B cells, mainly in patients that received manipulated dendritic cells. Detectable C-peptide in patients that had undetectable pretreatment C-peptide.	First use of tolerogenic dendritic cells in human autoimmunity.
Crohn’s disease			
2. Jauregui-Amezaga *et al* (2015)^102^ Phase I dose escalation study	9 patients with refractory Crohn’s disease received autologous monocyte-derived tolDC via sonography-guided intraperitoneal injections in six cohorts: a one-time injection of 2×10^6^/5×10^6^/10×10^6^ cells for the first 3 cohorts and three biweekly intraperitoneal injections at same escalating doses for another three cohorts.	No adverse effects were detected during tolDC injection or follow-up. Some anecdotal efficacy was observed and one patient achieved remission.	TolDC were not loaded with specific antigens. Three patients withdrew due to worsening symptoms.
Rheumatoid and inflammatory arthritis			
3. Benham *et al* (2015)^104^ Phase I randomised controlled study	34 patients with RA carrying HLA-DRB1 ‘shared epitope’ allele. 18 received autologous monocyte-derived tolDC intradermally at a dose of between 0.6 to 4.5×10^6^ cells (depending on yield) while 16 were controls	Well tolerated. Low grade adverse events including transient leucopoenia, anaemia and transaminitis. Treatment was associated with reduction in effector T cells and an increased regulatory:effector T cell ratio.	First use of dendritic cells for treatment of RA. TolDC were exposed to citrullinated peptides to confer antigen specificity
4. Bell *et al* (2016)^103^ Phase I unblinded randomised controlled dose escalation study	Monocyte-derived autologous tolDC. Three cohorts of patients with rheumatoid or other inflammatory arthritis received 1×10^6^, 3×10^6^, or 10×10^6^ cells into an inflamed knee. DC exposed to synovial fluid during culture as a source of auto-antigen. A fourth (control) cohort received arthroscopic washout alone.	Safe and acceptable procedure, feasible to manufacture tolDC from peripheral blood of patients with arthritis. Arthroscopically assessed synovial vascularity and synovitis improved in some patients who received TolDC.	First intra-articular administration of tolDC. No consistent immunomodulatory trend in peripheral blood between treatment and control groups. No evidence for DC-induced joint flare (indicating DC stability).

TolDC, tolerogenic dendritic cells.

A potential advantage of (autoantigen-loaded) tolDC compared with MSC is their capacity to specifically target autoreactive T cells, without non-specific immune suppression.^103 104^ Other similar antigen-specific cells are actively being investigated, especially in transplantation. These include regulatory macrophages (Mregs),^106–108^ myeloid derived suppressor cells^109^ and MSC-conditioned monocytes.^110^ While other applications remain preclinical, regulatory macrophages have been studied in humans in the context of renal transplantation. In a recent case report,^108^ two patients received donor-derived Mregs at doses of 7.1×10^6^ and 8×10^6^ cells/kg intravenously prior to receiving living donor renal transplants. Both patients were eventually weaned from steroids over 10 weeks leaving maintenance low dose tacrolimus. Transfused Mregs were shown to secrete IL-10 and suppress T cell proliferation by cell-cell contact and IFN-γ induced IDO activity.^108^ Both patients showed increased numbers of circulating Tregs post-transplant and a peripheral blood gene expression profile indicative of tolerance according to the Indices of Tolerance (IOT) research network.^111^

### Regulatory T cells

‘Natural’ CD4^+^CD25^+^FoxP3^+^ regulatory T cells (Tregs) play a central role in immune tolerance in health. While the evidence is not always definitive, Treg defects or deficiencies have been implicated in several autoimmune diseases.^47 112^ As with MSCs and DCs, considerable effort has therefore been dedicated to developing methodologies to isolate and expand these cells, as a potential tolerogenic therapy for autoimmune disease. Isolation uses the cell surface markers CD4, CD25 and usually CD127^low^. Subsequent expansion generally uses anti-CD3, anti-CD28 and IL-2 (figure 2). The expanded cells can, in theory, be rendered disease-specific by expansion in the presence of relevant autoantigens or genetic manipulation of TCR expression.^113^ Expanded Tregs have been used preclinically to treat murine models of autoimmunity, especially type 1 diabetes^114–118^ and, in some studies, Tregs were expanded with DCs to confer antigen specificity. In humans, early trials took place in patients with GVHD following bone marrow transplantation. For example, transfusion of HLA partially matched allogeneic umbilical cord blood derived Tregs at a dose of 0.1–30×10^5^ Treg/kg, following double umbilical cord blood transplantation, was associated with a reduced incidence of acute GVHD when compared with identically treated controls without Treg.^119^ Tregs have also been used in a phase I study to prevent GVHD by infusing donor-specific ex-vivo expanded Tregs prior to haploidentical haematopoietic stem cell transplantation without post-transplantation GVHD prophylaxis.^120^

The first description of expanded Treg administration in human autoimmunity was in children with type 1 diabetes.^121^ Ten children received intravenous injections of autologous Tregs in two dosing cohorts (10×10^6^ and 20×10^6^ cells/kg) and followed for 6 months (table 4). A matched control group was used to compare clinical improvement after infusion. The treatment group, on average, had lower insulin requirements at 6 months compared with their matched controls. In an extension of this study, a higher dose of up to 30×10^6^ cells/kg was well tolerated and associated with some clinical improvement after 12 months (reduction in insulin requirement and higher C-peptide levels).^122^ In a recent study in adults with newly diagnosed type 1 diabetes,^50^ a dose escalation protocol was used to assess the maximum tolerated dose of Tregs. Patients received intravenous infusions of Tregs up to a target dose of 2.3×10^9^ cells, experiencing no serious adverse effects. In vitro analysis showed that expansion of the Tregs increased the overall number of cells and their functional activity/potency. In this study, the DNA of expanded Tregs was labelled with deuterium, allowing in vivo tracking. Up to 25% of transfused Tregs survived in the peripheral blood after 1 year. Furthermore, deuterium did not appear in other lymphocyte populations suggesting expanded Tregs were stable after administration. Autologous Tr1 cells were also well tolerated when administered intravenously in 20 patients with Crohn’s disease with associated improvement in disease activity.^123^

**Table 4:** Clinical trials with expanded regulatory T cells (Tregs) in autoimmunity

Diseases and clinical trials	Number of patients, source of cells, dose and route of administration	Outcomes	Comments
**Ex-vivo expanded Tregs**			
Diabetes			
1. Marek-Trzonkowska *et al* (2012)^121^ Phase I non-randomised study	10 children with type 1 diabetes received autologous Tregs intravenously in two dosing cohorts (10×10^6^ and 20×10^6^ cells/kg body weight). A matched control group of 10 children did not receive a placebo. In the extension study,^122^ two extra patients were recruited for treatment, and 6 out of the total 12 patients received an additional infusion at 6–9 months (either 10×10^6^ or 20×10^6^ cells/kg) making up a total dose of 30×10^6^ cells/kg. Here, patients were followed up for 1 year.	No serious adverse events. Generally, treated children had lower insulin requirements at 6 months compared with matched controls, and recorded significantly higher c-peptide levels. A higher dose of 30×10^6^ cells/kg was also safely tolerated and was associated with better clinical outcomes (more patients in this group achieved remission, at 1 year with highest fasting and stimulated c-peptide levels and lowest HbA1C levels.	First in-human study of Tregs for autoimmunity
2. Bluestone *et al* (2015)^50^ Phase I dose-escalation study	14 adults with type 1 diabetes received intravenous autologous polyclonal Tregs in four dosing cohorts (0.056 to 23.5×10^8^ cells).	Safe. Transferred Tregs were long-lived and stable, with up to 25% surviving up to 1 year. Small sample size and heterogeneity of diabetes did not allow for efficacy assessment	Expanded Tregs had up to 4–8-fold higher suppressive activity than non-expanded Tregs from the same individual
Crohn’s disease			
3. Desreumaux *et al* (2012)^123^ Phase I/IIa multicentre study	20 patients with refractory Crohn’s disease received intravenously ovalbumin-specific Tr1 cells at 4 dose cohorts (10^6^, 10^7^, 10^8^, 10^9^ cells)	Safely tolerated with few adverse events. Clinical improvement with a reduction in Crohn’s disease activity index and inflammatory bowel disease questionnaires	First in human study of use of Tr1 cells for treatment of autoimmunity. Authors argue that ovalbumin is widely distributed in the GI tract and will activate Tr1 cells.
**In-vivo** **expanded Tregs**			
HCV-induced vasculitis			
4. Saadoun *et al* (2011)^134^ Phase I/IIa study in HCV-induced vasculitis	10 patients with HCV-induced vasculitis refractory to HCV therapy received 1.5×10^6^ IU subcutaneous (SC) IL-2 daily for 5 days, followed by three 5 day courses of 3×10^6^ IU/day at weeks 3, 6 and 9.	Safe with no major adverse events. There was a reduction in cryoglobulinaemia in 90% of patients and improvement in vasculitis in 80%. FoxP3^+^ Tregs also increased in peripheral blood.	Treatment did not induce effector T cell activation, vasculitis flare, or increased HCV viremia
Diabetes			
5. Long *et al* (2012)^135^ Phase 1 study in type 1 diabetes	9 patients with type 1 diabetes received 2–4 mg/day rapamycin for 3 months and 4.5×10^6^ IU IL-2 SC. thrice weekly for 1 month,	Safe with transient Treg increase in the first month but clinical and metabolic data showed worsening of β-cell function in all subjects.	No change in effector T cell frequencies but eosinophils and natural killer cells increased.
6. Hartemann *et al* (2013)^158^ Phase I/II randomised, double-blind placebo-controlled study in diabetes	24 patients with type 1 diabetes received either a placebo or one of three doses of IL-2. (0.33×10^6^ IU/day, 1×10^6^ IU/day or 3×10^6^ IU/day) SC for 5 days	Well-tolerated and few treatment related adverse events were reported (flu-like symptoms and injection site reactions). There was a significant dose-dependent increase in the proportion of Tregs in peripheral blood of patients.	
7. Todd *et al* (2016)^140^ Phase I/II non-randomised, open label, adaptive dose-finding trial	40 adults with type 1 diabetes received one injection of IL-2 SC in different dosing cohorts (0.04×10^6^ to 1.5×10^6^ IU/m^2^) and followed up for 7 days. The end point was the maximum percentage increase in Tregs (CD3^+^CD4^+^CD25^high^CD127^low^) from baseline frequency.	Well-tolerated. Optimum dose of IL-2 to induce 10% and 20% increases in Tregs were 0.101×10^6^ IU/m^2^ and 0.497×10^6^ IU/m^2^, respectively.	First adaptive dose-finding trial of IL-2 in diabetes.
8. Seelig *et al* (2017) 142 Phase I/II response-adaptive trial of repeat doses of IL-2 in diabetes	36 patients with type 1 diabetes received IL-2 at different dose-frequency combinations. Preliminary analysis of all accumulated data after completion of each cohort informed dose-frequencies of the following cohort. An initial learning phase involved 12 participants. Subsequent confirmatory cohorts were eight patients each.	Well tolerated apart from injection site reactions. The optimum regimen to maintain a steady state increase in Treg of 30% and CD25 expression of 25% without Teff expansion was 0.26×10 IU/m^2^ every 3 days.	Preprint data at the time of this review
ALOPECIA AREATA			
9. Castela *et al* (2014)^159^ Case series of low dose IL-2 in alopecia areata	5 patients received 1.5×10^6^ IU/day IL-2 SC for 5 days followed by 3 courses of 3×10^6^ IU/day at weeks 3, 6, and 9.	Safe with improvement in severity of alopecia tool (SALT) score (evaluated by two independent investigators). Significant increase in the number of Tregs was also seen in 80% of patients.	
SLE			
10. Humrich *et al* (2015)^137^ A case report of low-dose IL-2 in a patient with refractory SLE	1 patient received four treatment cycles of 1.5x10^6^ or 3x10^6^ IU IL-2 SC for five consecutive days with a washout period of 9–16 days after each course.	Clinical improvement was observed with reduction in anti-ds-DNA titre and SLEDAI score,	First evidence of possible therapeutic effect of low dose IL-2 in SLE.
11. von Spee-Mayer *et al* (2016)^136^ Phase I study in refractory SLE.	5 patients with refractory SLE were treated daily with 1.5×10^6^ IU IL-2 SC for five consecutive days	Safe with increased CD25 expression in Tregs and increased number of FoxP3^+^CD25^high^CD27^low^ Tregs during the treatment course.	
12. He *et al* (2016)^138^ Phase I study in active SLE	40 patients were treated with 3 courses of IL-2. Each course consisted of 1×10^6^ IU IL-2 SC alternate days for 2 weeks, with a 2 week drug-free period.	Treatment was safe and associated with a significant increase in CD25^high^CD127^low^ Tregs in the CD4^+^ T cell population. Significant clinical improvement was also observed such that up to 89.5% of patients had at least a 4-point decrease (SRI-4) in the SLEDAI after 12 weeks.	

IL, interleukin; SLE, systemic lupus erythematosus; SLEDAI, Systemic Lupus Erythematosus Disease Activity Index; UC, umbilical cord.

Concerns have been raised about the potential plasticity of Tregs in relation to their reliability as a cellular therapy. Natural Tregs form a relatively small proportion of peripheral blood CD4+ T cells and express no unique surface marker to facilitate their isolation. Nonetheless, enrichment of CD127^-/low^ cells generally suffices to minimise contamination with activated T cells. However, the propensity for expanded Tregs to express IL-17 was noted some years ago, with evidence suggesting that CD4^+^CD25^+^FoxP3^+^ Tregs can undergo transformation to pathogenic Th17 cells after repeated expansion.^124–126^ These studies demonstrated that epigenetic instability of the FoxP3 and retinoic acid receptor-related orphan receptor (RORC) loci accounted for the potential for Th17 (de-)differentiation. Further investigation demonstrated that both loci were stable in ‘naïve’ (CD45RA^+^) Tregs, when compared with memory (CD45RO^+^) Tregs.^126 127^ Therefore, use of CD45RA as an additional marker for Treg isolation should minimise expansion-induced epigenetic instability and produce a more homogenous tolerogenic Treg population, with low risk of Th17 transformation. In mice, evidence exists for cells that coexpress FoxP3 and RORγT, the murine equivalent of the Th17-lineage defining marker RORC.^128^ Despite a capacity to differentiate into either classical Tregs or Th17 cells, these cells demonstrated a regulatory function in murine diabetes.

The development of Tr1 cells as a therapy is at an earlier stage than regulatory T cell therapy. They can be expanded ex vivo from PBMC or CD4+ T cells. One method, using an IL-10 secreting DC (DC-10), can generate allospecific Tr1 cells for potential use in haematological or solid organ transplantation. An alternative technique generated ova-specific Tr1 cells for a phase 1b/2a clinical trial in Crohn’s disease.^123^

### In vivo expansion of regulatory T cells

IL-2 is a key cytokine for T cell activation and proliferation. Furthermore, because natural Tregs express high levels of CD25, the IL-2 receptor alpha chain, they are highly sensitive to stimulation by IL-2. In patients with cancer treated with peptide vaccine^129^ and DC-based vaccine immunotherapy,^130 131^ administration of IL-2 (with a rationale to expand effector T cells) actually led to in-vivo expansion of Tregs. This led to the theory that IL-2, particularly at low doses, will preferentially expand Tregs, informing preclinical experiments and clinical trials in autoimmunity. In a cohort of patients with chronic refractory GVHD, low dose IL-2 administration (0.3–1×10^6^ IU/m^2^) increased Treg:Teff ratio, with improvement in clinical symptoms and enabling tapering of steroid dose by a mean of 60%.^132^ Similarly, low dose IL-2 (1–2×10^5^ IU/m^2^) post-allogeneic SCT in children prevented acute GVHD when compared with those who did not receive low dose IL-2.^133^

Treatment of patients with Hepatitis C virus-induced, cryoglobulin-associated vasculitis with IL-2 at a dose of 1.5×10^6^ IU once a day for 5 days followed by 3×10^6^ IU for 5 days on weeks 3, 6 and 9 was associated with clinical improvement in 80% of patients as well as a reduction in cryoglobulinaemia and normalisation of complement levels.^134^ In a phase I trial in type 1 diabetes, administration of 2–4 mg/day of rapamycin and 4.5×10^6^ IU IL-2 thrice per week for 1 month led to a transient increase in Tregs but a paradoxical worsening of β-cell function, associated with an increase in circulating NK-cells and eosinophils.^135^ In SLE, a Treg defect associates with disease activity and appears secondary to defective endogenous IL-2 production.^136^ Exogenous low dose IL-2 appears to both reverse the biological defect and provide a potential therapeutic strategy.^136–138^

A common finding in trials of low dose IL-2 to treat autoimmunity is that effects are transient, declining once treatment is discontinued. Effects may not be limited to natural Tregs but also extend to FoxP3^+^CD8^+^ T cells, at least in type 1 diabetes.^139^ However, an optimum dosing regime is yet to be defined. Results from a recent adaptive dose-finding study in 40 patients with type 1 diabetes suggest that the optimal dose of a single injection of IL-2 that will induce 10% and 20% increases in Tregs over 7 days were approximately 0.10×10^6^ IU/m^2^ and 0.5×10^6^ IU/m^2^, respectively.^140^ This study also showed that the mean plasma concentrations of IL-2 at 90 min postinjection, even at the lowest doses, were higher than the hypothetical Treg-specific therapeutic window determined in vitro (0.015–0.24 IU/mL). This was associated with a dose-dependent transient desensitisation of Tregs (downmodulation of the beta subunit of IL-2 receptor (CD122)) and a decrease in the number of circulating Tregs and other lymphocytes, which improved 2 days after injection. These findings may explain the lack of response seen in some patients who have received daily injections of low-dose IL-2. A follow-on study by the same group investigated the optimum frequency of administration of IL-2 in type 1 diabetes.^141^ Results show that the optimum regimen to maintain a steady state increase in Treg of 30% and CD25 expression of 25% without Teff expansion was 0.26×10 IU/m^2^ every 3 days.^142^

It is unclear at this juncture whether in vivo expansion of Tregs might provide a superior therapeutic option in autoimmunity than ex vivo expansion and readministration. Conceivably the two modalities could be combined. Other attempts have been made to expand Tregs in vivo. One method is the administration of autoantigen in Freund’s incomplete adjuvant. In a phase I trial, a single dose of insulin-β-chain in IFA was administered intramuscularly to patients with type 1 diabetes.^143^ Treatment was well tolerated and appeared to stimulate robust antigen-specific regulatory T cell populations in the treatment arm up to 24 months, although there was no statistically significant difference in mixed meal stimulated c-peptide responses compared with the control group. Other methods are the probiotic use of whole helminths or their unfractionated products and administration of purified excretory/secretory helminths’ products. In preclinical studies using animal models of RA, MS, Crohn’s disease and type 1 diabetes, they induce Tregs (and other regulatory cells) in vivo and prevent autoimmunity.^144–146^ However, clinical trials are yet to show consistent encouraging results in humans.^145^

## Where are we now?

Results to date from human clinical trials have shown that cellular therapies are, at minimum, safe and feasible, and therefore worth exploring further in our pursuit of therapeutic tolerance induction. The regenerative properties of MSCs could additionally provide an element of tissue replenishment, repairing some of the damage that inevitably accompanies autoimmunity. However, most of the studies outlined in this review are at the very earliest phases of clinical development. Phase II and, ultimately, phase III studies will be needed to confirm their efficacy. Furthermore, as with any tolerogenic therapy in autoimmunity, clear objectives are required for efficacy trials. In transplantation, ‘operational tolerance’ is present when immunosuppression can be removed without allograft rejection. The situation is less clear in autoimmunity. Re-establishment of self-tolerance should equate with life-time drug-free remission, which has been demonstrated in some animal models when tolerogenic cells are administered both prophylactically and therapeutically.^42 95^ However, tolerance takes time to develop and tolerogenic therapies may not reduce symptoms in the short-term, necessitating the temporary continuation of more conventional therapies. Furthermore, immunosuppressive drugs and glucocorticoids could potentially interfere with tolerance induction as previously suggested for calcineurin inhibitors.^147^ Careful clinical trial designs will therefore be fundamental in order to identify, robustly, tolerance induction. In the short term, this is likely to require immune monitoring, for example, using autoantibody arrays and MHC-peptide tetramers, in order to track and interrogate the quality and quantity of the autoantigen-specific response.^148 149^ To date, cellular therapy trials have only occasionally incorporated experimental medicine end-points, for example, to measure longevity of cells, their distribution in vivo or to determine appropriate dosage.^123 140^ It is important that future trials adopt a similar philosophy, both to advance therapeutic development and also for ethical reasons.

Other factors to consider during the development of tolerogenic cellular therapies include the route of delivery. For more standard therapeutics, the main decision is usually oral vs parenteral delivery. For cellular therapies, the route has to be parenteral but the decision is potentially more sophisticated. For example, where might TolDC regulate an aberrant autoimmune response? In the target tissue, the draining lymph nodes, the central lymphoid organs? Route of delivery is likely to influence the therapy's ultimate destination, and treatment development needs to encompass work that demonstrates the cells express appropriate homing receptors. And then, there are the more standard developmental questions such as dosage and frequency of administration—a true tolerogenic therapy should only require a single ‘course’ of treatment but, in a patient with a propensity to autoimmunity, regular re-treatments may be required to keep autoreactivity at bay. Choice of autoantigen is also critical for certain cellular therapies. And last, cost-effectiveness has to be demonstrated for any novel treatment. However, the health economics would be very different for a tolerogenic therapy if it could truly avoid the need for chronic immunosuppressive therapy and its complications, not to mention the ravages of autoimmunity-associated tissue damage and comorbidities, such as cardiovascular disease.

The costs of isolating and expanding cells for therapy are significant but collaborations across academic research centres and commercial partners will solve some logistical challenges of clinical grade manufacture. Such challenges include cell source, cell isolation and expansion techniques, culture media and reagents, potency markers and genetic manipulation techniques where required (figure 2). These need to be standardised to ensure reproducibility because different cell manufacturing techniques will lead to subtle or even unidentified phenotypic differences in the final product. For example, it is unclear whether different types of tolDC, manufactured using distinct techniques, will have significantly different clinical effects.^150^ Measurement of potency is therefore a critical step prior to the release and administration of any cellular therapy product.^151^

At one point, the costs of cell manufacturing were envisaged to be a potential barrier to the development of immunomodulatory cell therapies. However, with the success of cellular therapeutics such as chimeric antigen receptor T cells for cancer, significant investment has been made in relevant technologies. For example, closed bioreactors can enable manufacture of large quantities of GMP-grade cells within a shorter period of time than labour-intensive, open culturing in flasks and bags.^152^ Such technologies are inherently adaptable, and therefore transferrable to different types of cellular therapy,^153^ helping to achieve cost-effectiveness and reducing batch-to-batch variability.

Eventually, and assuming positive results, comparative effectiveness trials across cell types (MSCs, TolDC and Tregs) may be required to determine which products are best suited for different forms and stages of autoimmunity. For example, MSCs, because of their regenerative capacity, may be favoured in conditions such as Crohn’s disease and MS where tissue regeneration would be advantageous. On the contrary, Tregs may be preferred in diseases with documented evidence of Treg dysfunction such as type 1 diabetes and SLE, because ex-vivo expansion of Tregs can reverse Treg dysfunction.^154^ The effects of different cell types is being investigated in transplantation in The ONE Study.^155^ In this collaborative study, different immunosuppressive cell populations (tolerogenic macrophages, myeloid derived suppressor cells, tolDC, monocytes conditioned by MSCs, IL-10 induced DCs and rapamycin-conditioned DCs) are manufactured from the same leukapheresis product, removing one element of variability when comparing these very different therapies. Cells are then studied in different disease contexts to determine the best approach to treatment. It may also prove possible to combine different cells to produce synergistic effects.

As tolerance can break down many years before the onset of clinical disease, it is also important to consider the optimal timing of cellular therapies. Detection of preclinical autoimmunity may provide a window of opportunity to treat and cure these diseases with safe interventions before symptom onset and before tissue damage has accrued. Epitope spreading, with broadening of the autoimmune repertoire alongside the non-specific effects of tissue damage, might render therapeutic tolerance induction more difficult in established disease, despite phenomena such as infectious tolerance and linked suppression.^156^ Appropriate immune monitoring will be even more important in disease, as a means to establish benefit in the absence of symptoms or signs. In-depth studies of allograft recipients who have achieved operational tolerance have identified biomarkers that appear specific for the tolerant state. These may be useful for monitoring attempts at tolerance induction prospectively.^157^

## Conclusion

It is an exciting time for tolerogenic cellular therapies. Rapid advances can be expected in the short to medium term catalysed by progress in manufacturing technologies, advances in the development of immune monitoring techniques and the identification of tolerance biomarkers, alongside an acceptance that earlier treatment may be ethically justified if the therapeutic target is tolerance induction. Whether any, or all, of the cells discussed in this review will ultimately demonstrate robust tolerogenic effects must await formal clinical trials of efficacy; and we should be as certain as we can be that the timing, route and dosing of therapy is optimal before conducting the ‘definitive’ studies. These are not easy challenges but they are tractable and, currently, there is a large amount of intellectual energy directed at solving them.
